# Exposure–safety analyses of nintedanib in patients with chronic fibrosing interstitial lung disease

**DOI:** 10.1186/s12890-021-01598-0

**Published:** 2021-07-21

**Authors:** Ulrike Schmid, Benjamin Weber, Celine Sarr, Matthias Freiwald

**Affiliations:** 1 Translational Medicine and Clinical Pharmacology, Boehringer Ingelheim Pharma GmbH & Co. KG, Birkendorfer Strasse 65, 88397 Biberach an der Riss, Germany; 2 Pharmetheus AB, Uppsala, Sweden

**Keywords:** Nintedanib, Idiopathic pulmonary fibrosis, Systemic sclerosis-associated interstitial lung disease, Progressive fibrosing ILDs, Exposure–safety relationship, Liver enzyme elevation

## Abstract

**Background:**

Nintedanib reduces the rate of decline in forced vital capacity in patients with idiopathic pulmonary fibrosis (IPF), other chronic fibrosing interstitial lung diseases (ILDs) with a progressive phenotype and systemic sclerosis-associated ILD (SSc-ILD). The recommended dose of nintedanib is 150 mg twice daily (BID).

**Methods:**

Data from Phase II and III trials in IPF and Phase III trials in SSc-ILD and progressive fibrosing ILDs other than IPF were analyzed to investigate the relationship between nintedanib plasma concentrations (exposure) and safety (liver enzyme elevations [defined as transaminase elevations equal or greater than 3 times the upper limit of normal] and diarrhea).

**Results:**

Using data from 1403 subjects with IPF treated with 50–150 mg nintedanib BID, a parametric time-to-first-event model for liver enzyme elevations was established. Besides exposure, gender was a significant covariate, with a three–fourfold higher exposure-adjusted risk in females than males. Subsequent analysis of combined data from IPF, SSc-ILD (n = 576) and progressive fibrosing ILD (n = 663) studies suggested a consistent exposure–liver enzyme elevation relationship across studies. No exposure–diarrhea relationship was found using data from the various fibrosing ILDs, but diarrhea risk was dependent on dose administered.

**Conclusions:**

The positive correlation between exposure and risk of liver enzyme elevations was consistent across nintedanib studies in IPF, SSc-ILD and progressing fibrosing ILDs other than IPF. The effect size does not warrant a priori dose adjustment in patients with altered plasma exposure (excluding hepatic impairment patients, where there are specific labelling recommendations). For diarrhea, dose administered was a better predictor than exposure.

**Supplementary Information:**

The online version contains supplementary material available at 10.1186/s12890-021-01598-0.

## Introduction

Nintedanib is a tyrosine kinase inhibitor that inhibits key pathways involved in lung fibrosis in interstitial lung diseases (ILDs) and is approved for the treatment of idiopathic pulmonary fibrosis (IPF), other chronic fibrosing interstitial lung diseases with a progressive phenotype (progressive fibrosing ILDs) and for the treatment of systemic sclerosis-associated ILD (SSc-ILD) [1, 2]. In the pivotal INPULSIS-1^®^ and INPULSIS-2^®^ trials and a supportive Phase II dose finding trial (TOMORROW^®^) in patients with IPF, a twice daily (BID) 150 mg dose of nintedanib significantly reduced the annual rate of decline in forced vital capacity (FVC) suggesting slowing in disease progression [3–6]. Thereafter, clinical efficacy of the 150 mg dose has additionally been shown in randomized trials in SSc-ILD [7] and progressive fibrosing ILDs other than IPF [8]. In clinical trials, side effects of nintedanib were generally manageable, while common adverse events (AEs) in the nintedanib arms were gastrointestinal (mainly diarrhea, nausea and vomiting) and hepatic enzyme elevations. Recommendations for monitoring and dose modification for management of AEs are included in the nintedanib prescribing information [2].

The pharmacokinetics of nintedanib and factors affecting its plasma concentration (exposure) have been described previously [9–12]. Gender and renal function have no influence on nintedanib pharmacokinetics, whereas hepatic impairment, Asian race, body weight and age affect nintedanib exposure [10, 12]. Other than hepatic impairment, these covariates have a relatively small effect on plasma exposure, whereas unexplained interpatient variability in nintedanib exposure is high [10]. For patients with mild and moderate hepatic impairment, an approximately twofold and eightfold increase in nintedanib exposure has been shown respectively [12]. In this analysis, we explore the association between nintedanib plasma exposure and safety—specifically diarrhea and liver enzyme elevations—and evaluate the impact of selected intrinsic and extrinsic factors on these outcomes. The models were first developed in IPF with different dose levels available; subsequently, data from patients with SSc-ILD and progressive fibrosing ILDs other than IPF randomized to the therapeutic dose of 150 mg BID or placebo were used to further explore associations between plasma exposure and safety.

## Materials and methods

### Studies included

Data were analyzed from 1403 subjects with IPF treated with 50–150 mg nintedanib BID (n = 895) or placebo (n = 508) in three clinical studies: one Phase II trial (TOMORROW) evaluating 50–150 mg nintedanib BID or placebo (n = 342) [6], and two identical Phase III trials (INPULSIS-1 and INPULSIS-2) evaluating 150 mg nintedanib BID or placebo (n = 1061) [3].

In addition, data from 576 subjects with SSc-ILD randomized to either 150 mg nintedanib BID (n = 288) or placebo (n = 288) in the SENSCIS^®^ Phase III trial [7] and from 663 subjects with chronic fibrosing ILDs with a progressive phenotype other than IPF randomized to either 150 mg nintedanib BID (n = 332) or placebo (n = 331) in the INBUILD^®^ Phase III trial [8] were analyzed. Study designs and results have been published previously [3, 6–8, 13–15]. In all studies, dose interruption or reduction for the management of AEs was allowed. In the INBUILD study, specific efforts were made to exclude patients with IPF (as efficacy and safety had already been established for IPF).

The primary efficacy endpoint in all studies was the annual rate of decline in FVC, as assessed over a 52-week treatment period (despite a variable treatment period beyond week 52 in the SENSCIS and INBUILD trials).

Safety was assessed based on the occurrence of AEs, laboratory tests, physical examination, vital sign recordings, and 12-lead electrocardiogram.

For the assessment of nintedanib plasma exposure, at least two pre-dose blood samples were scheduled (Table 1) and analyzed by validated liquid chromatography mass spectrometry.

**Table 1:** Summary of the trials contributing data to the analyses

Trial	Population	Treatments	PK sampling	Liver enzyme assessment^a^
TOMORROW Randomized Phase II, 52 weeks	IPF (n = 342)^b^	Placebo (n = 85)	Pre-dose, Days 1, 29, 169, 365 and end of treatment	Baseline, Days 1, 15, 29, 43, 85, 127, 169, 211, 253, 309, 365
		Nintedanib 50 mg BID (n = 86)		
		Nintedanib 100 mg BID (n = 86)		
		Nintedanib 150 mg BID (n = 85)		
INPULSIS-1 Randomized Phase III, 52 weeks	IPF (n = 513)	Placebo (n = 204)	Days 29 and 169	Baseline, Days 1, 15, 29, 43, 85, 127, 169, 211, 253, 309, 365
		Nintedanib 150 mg BID (n = 309)		
INPULSIS-2 Randomized Phase III, 52 weeks	IPF (n = 548)	Placebo (n = 219)	Days 29 and 169	Baseline, Days 1, 15, 29, 43, 85, 127, 169, 211, 253, 309, 365
		Nintedanib 150 mg BID (n = 329)		
SENSCIS Randomized Phase III, 52 weeks	SSc-ILD (n = 576)	Placebo (n = 288)	Days 29 and 169	Baseline, Days 1, 15, 29, 43, 85, 127, 169, 211, 253, 309, 365
		Nintedanib 150 mg BID		
		(n = 288)		
INBUILD Randomized Phase III, 52 weeks	Progressive fibrosing ILD (n = 663)	Placebo (n = 331)	Days 29 and 169	Baseline, Days 1, 15, 29, 43, 85, 127 (optional), 169, 211 (optional), 253, 309 (optional), 365
		Nintedanib 150 mg BID (n = 332)		

*BID* twice daily, *ILD* interstitial lung disease, *IPF* idiopathic pulmonary fibrosis, *PK* pharmacokinetic, *SSc* systemic sclerosis

^a^Treatment period beyond week 52 in the SENSCIS and INBUILD trials was not used for analysis

^b^From 428 patients treated in the TOMORROW trial, 342 patients are shown (86 patients randomized to 50 mg nintedanib once daily were excluded from analysis)

The studies were conducted in accordance with the Declaration of Helsinki and approved by the ethics committee of the co-ordinating centre: Comitado Etico Provinciale, Modena, Italy (TOMORROW and INPULSIS-1); Committee on Human Research, UCSF, San Francisco CA, USA (INPULSIS-2); Kantonale Ethikkommision (SENSCIS); Pulmonary and Critical Care Medicine Chesapeake Institutional Review Board, Columbia, USA (INBUILD). Written informed consent was obtained from all subjects before study entry. A summary of the trials contributing data to the analyses is shown in Table 1.

### Safety endpoints and exposure metrics

For the derivation of the safety endpoints, treatment-emergent AEs or laboratory values with an onset/worsening date between the first drug intake and a residual effect period after the discontinuation of study medication (14 days for the Phase II TOMORROW trial and 28 days for Phase III trials) within a 52-week treatment period were considered. The following safety endpoints were derived: (1) time of first alanine transaminase (ALT) and/or aspartate transaminase (AST) elevation to at least three times the upper limit of normal (ULN) over 52 weeks; and (2) time of first onset of diarrhea of any grade over 52 weeks.

Observed and population pharmacokinetic (PopPK) model-predicted pre-dose drug concentrations at steady state (C_pre,ss_) were used as exposure metrics for IPF. For the derivation of observed C_pre,ss_, the available pre-dose plasma measurements from each patient were collapsed into one value (geometric mean of all dose-normalized pre-dose concentrations per patient; in case of one value, this value was taken) and multiplied by the starting dose. To obtain predicted C_pre,ss_ values, empirical Bayes estimates were generated using the PopPK model in IPF [10, 16] and observed nintedanib concentrations as well as baseline patient characteristics of relevant covariates in the respective trials. If no valid nintedanib concentration was available for a particular patient, only the baseline patient characteristics were considered for the prediction. The dose-normalized predicted C_pre,ss_ concentrations were multiplied by the actual single dose taken by a patient on a specific day to account for dose reductions and treatment interruptions (use of time-matched exposure for predicted C_pre,ss_). As the two exposure measures provided comparable results in the analyses of IPF, only predicted C_pre,ss_ concentrations were used for subsequent exposure-safety analyses in patients with SSc-ILD and progressive fibrosing ILDs other than IPF (see “Discussion” section). The Phase II TOMORROW trial also included an arm with patients treated at 50 mg nintedanib once daily (n = 86), which was excluded from the current analysis. This was due to the exposure measure of C_pre,ss_ referring to one specific time point to represent the overall plasma exposure of a patient in the exposure-safety analysis. Depending on the influence of other pharmacokinetic parameters on response (e.g. maximum plasma levels were expected to be higher for the once-daily schedule than for the BID schedule despite providing the same C_pre,ss_), steady-state trough concentrations from daily administration were not considered subject to the same interpretation as those from BID administration.

### Exposure–liver enzyme elevations analyses

An exposure–liver enzyme elevation model was initially developed using data from combined IPF studies. Parametric time-to-first-event (survival) modelling [17] was applied to investigate the relationship between nintedanib exposure and the probability of developing a liver enzyme elevation. The model was first fit to placebo-treated patients, where survival times were assumed to follow a Weibull distribution [17]. This model was compared with a model assuming a constant baseline hazard. Subsequently, the nintedanib drug effect was included by simultaneously analyzing all patients (both placebo- and nintedanib-treated). Linear, log-linear, maximum effect (E_max_) and sigmoidal E_max_ relationships on the log of the hazard were evaluated as drug effect functions, and both PopPK-predicted and observed C_pre,ss_ values were used as exposure measures.

Finally, a stepwise covariate analysis consisting of univariate analysis (*p* = 0.05), forward inclusion (*p* = 0.05) and backward elimination (*p* = 0.001) was used to explore factors potentially influencing the exposure–safety relationship. Stricter criteria were used in the backward elimination step, as commonly done to reduce selection bias of the multiple covariate testing procedure [18]. The covariates tested for IPF included age, height, gender, body weight, body surface area, Asian subpopulations, smoking status and study.

The exposure–liver enzyme elevation model developed for IPF was subsequently applied to combined data from the IPF Phase II/III studies (TOMORROW, INPULSIS-1, INPULSIS-2) and the SENSCIS Phase III trial. Finally, data from the INBUILD trial were added (pooled analysis of the TOMORROW, INPULSIS-1, INPULSIS-2, SENSCIS and INBUILD trials).

The drug effect was re-evaluated by exploring linear, log-linear and E_max_ functions on the log of the hazard. Covariates were assessed using a stepwise modelling approach as described for IPF. SSc subtype (diffuse cutaneous SSc vs. limited cutaneous SSc), anti-topoisomerase antibody status (positive vs. negative) and mycophenolate (mofetil/sodium/acid) use at baseline were explored as additional SSc-ILD-specific covariates besides the demographics typically investigated for IPF. In addition, methotrexate use at baseline (yes vs. no), use of disease-modifying antirheumatic drugs with known hepatotoxic effects at baseline (yes vs. no) and FVC % predicted at baseline (as a surrogate of disease severity) were explored as covariates based on the combined data across all indications. Differences between study populations (IPF studies vs. SENSCIS vs. INBUILD) were also explored.

For the definition of liver enzyme elevation events in the INBUILD trial, two different reference ranges of liver enzyme (ALT and AST) measurements were considered. The reference ranges for ALT and AST as defined in 2014 by the central laboratory provider were used for the primary analysis of the INBUILD trial and are hereinafter referred to as “2014 reference ranges used for INBUILD primary analysis”. Independently from the INBUILD trial, the central laboratory provider updated the reference ranges for the applied ALT and AST assays in 2019, in parallel to the study conduct, to better align ranges with usual and customary practice. These are hereinafter referred to as “2019 updated reference ranges” (see Additional file 1: Table S1). The update was triggered by consultation with different peer reference laboratories and recent literature [19] and was based on a reference population of 256 male or female volunteers. A sensitivity analysis was conducted with the 2019 updated reference ranges for ALT and AST to assess the potential influence of this update.

### Exposure–diarrhea analyses

Congruent to the analyses focusing on liver enzyme elevations, exposure–diarrhea analyses were initially developed based on data from IPF studies. Parametric time-to-first-event (survival) modelling was applied to investigate the relationship between nintedanib exposure and the probability of developing an episode of diarrhea (any severity grade) over 52 weeks. As with the exposure–liver enzyme elevation analyses, the analysis consisted of three steps: (1) time-to-first-event analysis based on placebo-treated patients; (2) addition of the effect of nintedanib exposure on the risk of experiencing diarrhea (using observed and predicted C_pre,ss_) by simultaneously fitting placebo- and nintedanib-treated patients; and (3) a stepwise covariate analysis [18] consisting of univariate analysis (*p* = 0.05), forward inclusion (*p* = 0.05) and backward elimination (*p* = 0.001). Linear, log-linear, E_max_ and sigmoidal E_max_ relationships were evaluated as drug effect functions. The same covariates as for the liver enzyme elevation variable were evaluated. However, as no exposure–diarrhea model could be established to describe the data, the covariate analysis was additionally performed for a model using categorical dose (instead of plasma exposure) as a predictor of the diarrhea risk.

Exploratory analyses were performed to further differentiate between exposure and dose as predictors of the diarrhea risk. Therefore, patients from the 100 mg BID treatment group were optimally matched by nintedanib exposure to patients from the 150 mg BID treatment group (1:2 matching) using a SAS^®^ macro developed by Bergstralh and Kosanke [20]. Afterwards, the number of diarrhea events was compared between the exposure-matched treatment groups (including patients with comparable plasma exposure despite having received different nintedanib doses). Exposure–diarrhea modelling was not further pursued for data from trials in SSc-ILD (SENSCIS) and chronic fibrosing ILDs with a progressive phenotype other than IPF (INBUILD). Instead, exploratory analyses across trials evaluating the number and proportion of diarrhea events by exposure tertile and severity grade were performed.

### Model selection and evaluation

Model selection was guided by numerical change in objective function values; identifiability of parameters and precision of parameter estimates; the correlation between the estimates of fixed-effect parameters; numerical stability; ability to obtain a successful covariance step; and visual inspection of basic goodness-of-fit plots. Adequacy of the base and final models was confirmed using simulation-based diagnostics and bootstrap analysis [21].

### Software

The exposure–safety analyses were performed using NONMEM^®^ (version 7.3 or higher, ICON Development Solutions, Hanover, MD, USA). The maximal-likelihood estimation method ($ESTIMATION METHOD = 0 LIKE) was used for model fitting and parameter estimation in the time-to-first-event modelling.

Visual predictive checks, non-parametric bootstrap analysis and covariate analysis were performed using Perl-speaks-NONMEM (version 4.6.0 or higher) [22, 23]. Post-processing and descriptive statistics were performed using R (version 3.2.2 or higher) and SAS^®^ (version 9.2 or higher; SAS Institute Inc, Cary, NC, USA).

## Results

### Description of data set

A total of 2642 patients with IPF, SSc-ILD or chronic fibrosing ILDs with a progressive phenotype other than IPF from the TOMORROW, INPULSIS, SENSCIS and INBUILD trials were considered for exposure–safety analyses; 1403 patients with IPF who received 50–150 mg nintedanib BID (n = 895) or placebo (n = 508) in TOMORROW and INPULSIS were used for initial model development. Subsequently, 576 patients with SSc-ILD from the SENSCIS trial, randomized to either 150 mg nintedanib BID (n = 288) or placebo (n = 288), and 663 patients with progressive fibrosing ILD other than IPF randomized to either 150 mg nintedanib BID (n = 332) or placebo (n = 331) from the INBUILD trial, were included. Baseline demographic characteristics are given in Table 2.

**Table 2:** Demographic characteristics

Characteristic	TOMORROW (IPF) n = 342^a^	INPULSIS-1 (IPF) n = 513	INPULSIS-2 (IPF) n = 548	SENSCIS (SSc-ILD) n = 576	INBUILD (Progressive fibrosing ILD) n = 663
Age, years	65 (8.4)	67 (8.3)	67 (7.8)	54 (12.2)	66 (9.8)
Body weight, kg	77 (14.7)	82 (16.6)	77 (16.1)	70 (15.9)	77 (17.4)
Height, cm	167 (9.0)	169 (8.9)	167 (9.4)	164 (9.8)	165 (10.0)
FVC, % predicted	82 (17.9)	80 (17.1)	79 (18.4)	73 (16.7)	69 (15.6)
Race, n (%)					
White	270 (79)	333 (65)	275 (50)	387 (67)	488 (74)
Black	0	0	2 (< 1)	36 (6)	10 (2)
Asian	72 (21)	106 (21)	214 (39)	143 (25)	163 (25)
American Indian/Alaska native	0	1 (< 1)	1 (< 1)	5 (1)	0
Hawaiian/Pacific Islander	0	0	0	1 (< 1)	1 (< 1)
Multiple	0	0	0	4 (< 1)	1 (< 1)
Missing	0	73 (14)^b^	56 (10)^b^	0	0
Region, n (%)					
Asia	73 (21)	97 (19)	225 (41)	130 (23)	155 (23)
Europe	202 (59)	288 (56)	187 (34)	266 (46)	301 (45)
North America	10 (3)	70 (14)	104 (19)	142 (25)	136 (21)
Rest of the world	57 (17)	58 (11)	32 (6)	38 (7)	71 (11)
Underlying diagnosis (INBUILD STUDY), n (%)					
HP	–	–	–	–	173 (26)
iNSiP	–	–	–	–	125 (19)
Unclassifiable IIP	–	–	–	–	114 (17)
Autoimmune ILDs	–	–	–	–	170 (26)
Other ILDs	–	–	–	–	81 (12)

Data are presented as mean (SD) unless otherwise stated

*FVC* forced vital capacity, *HP* hypersensitivity pneumonitis, *IIP* idiopathic interstitial pneumonia, *ILD* interstitial lung disease, *iNSiP* idiopathic nonspecific interstitial pneumonia, *IPF* idiopathic pulmonary fibrosis, *SD* standard deviation, *SSc* systemic sclerosis

^a^From 428 patients treated in the TOMORROW trial, 342 patients are shown (86 patients randomized to 50 mg nintedanib once daily were excluded from analysis)

^b^Due to local laws, race information was not collected for some patients with study site located in France

Out of 2642 patients, 1194 (261 in the placebo groups and 933 in the nintedanib BID treatment groups) had a diarrhea event over 52 weeks, with 62–76% of patients experiencing diarrhea at the therapeutic dose of 150 mg BID and 18–32% of patients having diarrhea events in the placebo arms across different studies (Table 3). The number of patients with liver enzyme elevation events (ALT or AST elevations ≥ 3 × ULN) was low (106/2642 patients). A higher frequency of liver enzyme elevations was observed in patients in both arms of the INBUILD trial (2% placebo, 13% nintedanib) compared with the TOMORROW, INPULSIS and SENSCIS studies (1% placebo, 5% nintedanib), although the INBUILD rate was substantially lowered by using the 2019 updated reference ranges for ALT and AST measurements as evaluated in the sensitivity analysis (1% placebo, 8% nintedanib).

**Table 3:** Patients with on-treatment diarrhea events of any severity and elevations in liver enzymes over 52 weeks in nintedanib trials TOMORROW, INPULSIS, SENSCIS and INBUILD

Trials (population)	Treatment group	Diarrhea, n (%)	Liver enzyme elevations, n (%)^a^
TOMORROW and INPULSIS-1/2 (IPF)	Placebo	91 (18)	3 (1)
	Nintedanib 50 mg BID	17 (20)	2 (2)
	Nintedanib 100 mg BID	32 (37)	0 (0)
	Nintedanib 150 mg BID	445 (62)	36 (5)
SENSCIS (SSc-ILD)	Placebo	91 (32)	2 (1)
	Nintedanib 150 mg BID	218 (76)	14 (5)
INBUILD (progressive fibrosing ILD other than IPF)	Placebo	79 (24)	6 (2) *4 (1)*^a^
	Nintedanib 150 mg BID	221 (67)	43 (13) *26 (8)*^a^
**All**		**1194 (45)**	**106 (4) *87 (3)***^**a**^

The bold signifies the total amount

*ALT* alanine transaminase, *AST* aspartate transaminase, *BID* twice daily, *ILD* interstitial lung disease, *IPF* idiopathic pulmonary fibrosis, *SSc* systemic sclerosis

^a^For the INBUILD study, liver enzyme elevations are given based on the 2014 reference ranges for ALT and AST measurements used for the INBUILD primary analysis. The values according to the 2019 updated reference ranges used for sensitivity analysis (more closely aligned to the reference ranges used in the TOMORROW, INPULSIS and SENSCIS trials) are given in italics (see “Materials and methods” section and Additional file 1: Table S1)

### Exposure–liver enzyme elevation analyses

For the initial analyses based on IPF data, the number of patients experiencing an ALT and/or AST elevation ≥ 3 × ULN across the treatment groups was low (38 patients with events out of 895 nintedanib-treated patients and only 3 in the placebo group). Therefore, a parametric time-to-event model could not be fitted to the data from placebo patients, even if the model was reduced to a constant hazard model. By analyzing placebo and nintedanib-treated patients, a Weibull baseline hazard with a log-linear drug effect relationship between observed or predicted C_pre,ss_ and the log of the hazard showed the best model performance (in comparison to a linear, E_max_ or sigmoidal E_max_ function considering objective function values and parameter precision). Gender had a significant influence on the risk of developing liver enzyme elevations (independent of exposure), with females having an approximately three–fourfold higher exposure-adjusted risk than males. Although this trend was seen in both C_pre,ss_ models, gender remained significant after the final step of covariate analysis only in the predicted C_pre,ss_ model. Due to the consistent trend in visual predictive checks indicating a gender effect despite the limited number of events, this effect was retained in both models. Univariate analysis also identified patients with low height, low body weight or low body surface area as having a significantly higher risk of a transaminase elevation. However, after adjusting for gender, these covariates no longer had a significant effect on the exposure–safety relationship. Parameter estimates of the final model in IPF based on observed and predicted C_pre,ss_ are shown in Additional file 1: Table S2.

The exposure–liver enzyme elevation model developed in IPF was used as a starting point for analyses with pooled data from the IPF trials, SENSCIS and INBUILD. The model structure implemented for IPF was still found to be superior in terms of data fit and precision of parameter estimates as compared with other models (when re-assessing a linear drug effect function or an E_max_ function). Of the tested covariates, gender was confirmed as a significant covariate, as already observed based on IPF data only, with females estimated on average to have a 3.7-fold higher estimated exposure-adjusted risk of experiencing liver enzyme elevations than males. No significant difference in the exposure–safety relationship was detected between the IPF trials and the SENSCIS trial. A study effect for the INBUILD trial was, however, identified indicating a higher probability of experiencing liver enzyme elevations than for patients from the IPF trials or from the SENSCIS trial. No other significant covariate effects were detected (Additional file 1: Tables S3–S5).

In the subsequent sensitivity analysis, the final exposure-liver enzyme elevation model was applied to the reference ranges for ALT and AST in INBUILD as updated in 2019 (see “Materials and methods” section). In contrast to the model using the reference ranges from 2014, the 95% confidence interval (CI) of the study effect estimate in this model included the no effect level corresponding to a value of zero (95% CI − 0.109, 0.286), and the *p* value of the corresponding log likelihood test for significance of the effect was 0.36 (dOFV of 0.83 points, χ^2^ distribution); in other words, a difference in the exposure–liver enzyme elevation relationship was no longer found*.* Parameter estimates of the final liver enzyme elevation model, based on pooled data from the TOMORROW, INPULSIS, SENSCIS and INBUILD trials, are shown in Table 4 together with the results from sensitivity analysis. Visual predictive checks for the final exposure–liver enzyme elevation model are provided in Additional file 1: Figure S1, and indicate that this model described the observed data of all study populations well.

**Table 4:** Parameter estimates of the final exposure–liver enzyme elevations model for nintedanib using pooled data from clinical trials in IPF, SSc-ILD and progressive fibrosing ILD other than IPF (based on 2014 reference ranges for ALT and AST used for the primary INBUILD analysis) together with parameter estimates from sensitivity analyses (based on 2019 updated reference ranges for ALT and AST in INBUILD, with and without inclusion of study effect)

Parameter	Unit	Estimate (%RSE)
Final exposure–liver enzyme elevations model (use of 2014 reference ranges of ALT and AST for INBUILD)		
Scale factor λ (Weibull distribution)	1/day	0.000994 (37.9)
Shape factor γ (Weibull distribution)		0.371 (11.2)
Log-linear coefficient of drug effect_male,TOMORROW,INPULSIS,SENSCIS_		0.579 (21.8)
Gender on log-linear coefficient_female_		0.668 (34.1)
Study on log-linear coefficient_INBUILD_		0.328 (36.6)
Sensitivity analysis (use of 2019 updated reference ranges of ALT and AST for INBUILD)		
Scale factor λ (Weibull distribution)	1/day	0.000748 (42.8)
Shape factor γ (Weibull distribution)		0.382 (12.3)
Log-linear coefficient of drug effect_male,TOMORROW,INPULSIS,SENSCIS_		0.620 (24.0)
Gender on log-linear coefficient_female_		0.729 (37.7)
Study on log-linear coefficient_INBUILD_		0.0885 (114)
Sensitivity analysis (use of 2019 updated reference ranges of ALT and AST for INBUILD and study effect removed)		
Scale factor λ (Weibull distribution)	1/day	0.000733 (43.0)
Shape factor γ (Weibull distribution)		0.382 (12.3)
Log-linear coefficient of drug effect_male,TOMORROW,INPULSIS,SENSCIS,INBUILD_		0.640 (23.4)
Gender on log-linear coefficient_female_		0.741 (37.2)

Final exposure–liver enzyme elevation model (main analysis): 2642 subjects, 2642 observations, objective function of 1194.62; Sensitivity analysis with study effect: 2642 subjects, 2642 observations, objective function of 1039.04; Sensitivity analysis without study effect: 2642 subjects, 2642 observations, objective function of 1039.87 $h\left(t\right)=\lambda *f\left(Cpre,ss\right)*\gamma *{t}^{\gamma -1}$ $f\left(Cpre,ss\right)= {e}^{PH\_Cpre*\mathrm{log}\left(1+Cpre,ss\right)*(1+P{H}_{Gender})*(1+PH\_STUDY)}$

λ, scale parameter (Weibull distribution); γ, shape parameter (Weibull distribution); ALT, alanine transaminase; AST, aspartate transaminase; C_pre,ss_, pre-dose drug concentration in plasma at steady state; h(t), hazard at time t; IPF, idiopathic pulmonary fibrosis; PH_C_pre_, log-linear coefficient of the drug effect; PH_Gender_, gender effect on log-linear coefficient referring to females (set to 0 for males); PH_Study, study effect on log-linear coefficient referring to the INBUILD study (set to 0 for TOMORROW, INPULSIS and SENSCIS; in the sensitivity model without study effect, it is set to 0 for all studies); RSE, relative standard error (derived from standard errors provided by NONMEM); SSc-ILD, systemic sclerosis-associated interstitial lung disease

**Fig. 1:**
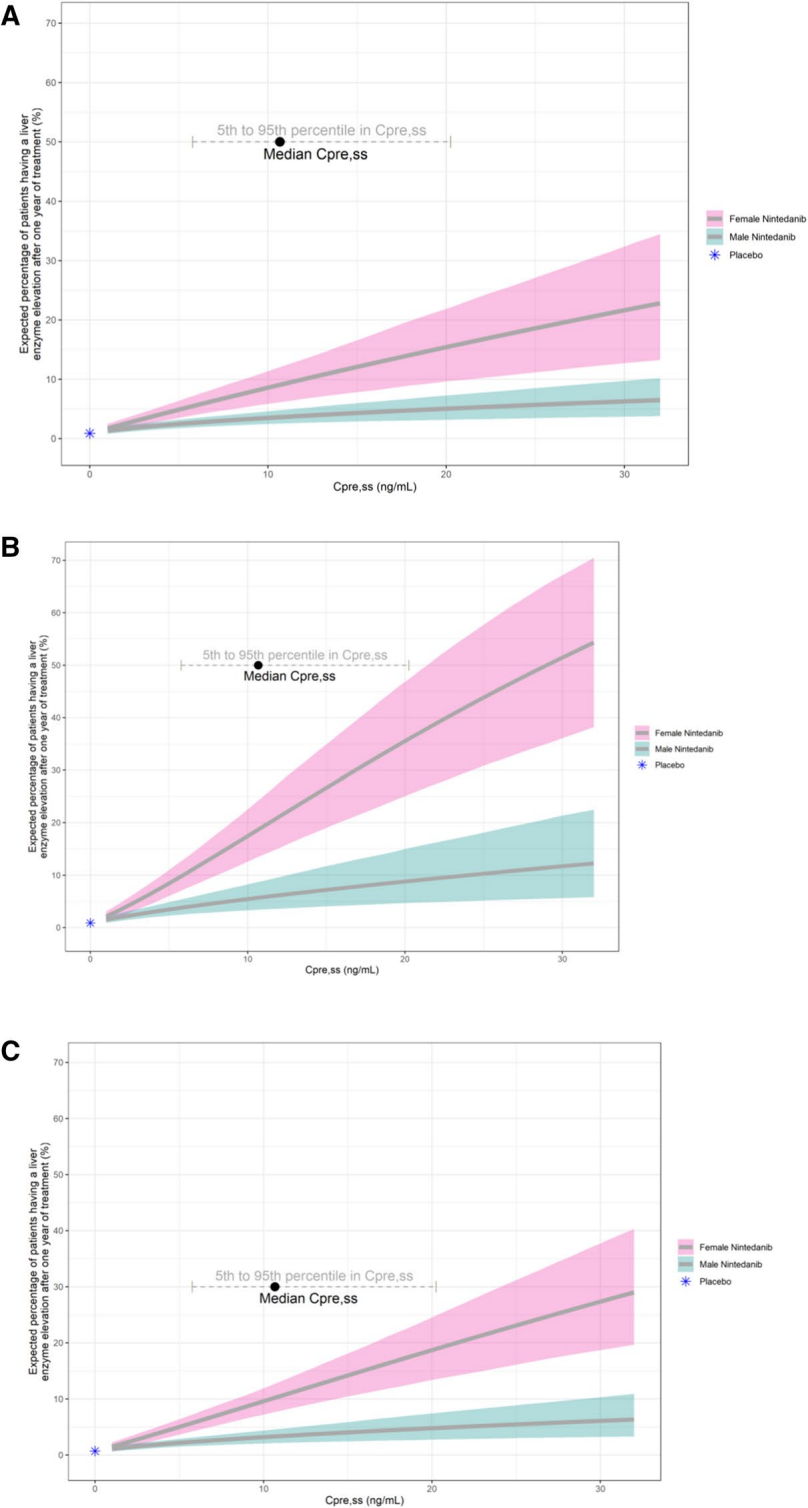
Expected percentage of patients having a liver enzyme elevation for different nintedanib plasma exposure levels (C_pre,ss_) after 1 year of treatment based on the final exposure–liver enzyme elevation model, using pooled data from trials in IPF, SSc-ILD and progressive fibrosing ILD other than IPF (TOMORROW, INPULSIS, SENSCIS and INBUILD). **A**, **B** Final liver enzyme elevation model based on 2014 references ranges of ALT and AST used for the INBUILD primary analysis. The figure is stratified by combined IPF and SSc-ILD trials (TOMORROW, INPULSIS, SENSCIS) versus the trial in progressive fibrosing ILDs other than IPF (INBUILD) **C** Sensitivity analysis with 2019 updated reference ranges of ALT and AST in the INBUILD trial (study effect removed). The solid lines represent the expected percentage of patients with liver enzyme elevations based on point estimates of the final liver enzyme elevation model. The shaded areas represent the 95% CI based on 2000 bootstrap replicates. The black-filled circle indicates the median C_pre,ss_ in patients receiving 150 mg nintedanib BID in TOMORROW, INPULSIS-1/2, SENSCIS and INBUILD. The dashed grey line indicates the 5th and 95th percentiles of C_pre,ss_. **A** Final exposure–liver enzyme elevation model: IPF and SSc-ILD trials (TOMORROW, INPULSIS, SENSCIS), **B** Final exposure–liver enzyme elevation model: progressive fibrosing ILD trial (INBUILD). **C** Sensitivity analysis (use of 2019 updated reference ranges of ALT and AST for INBUILD and study effect removed): trials in IPF, SSc-ILD and progressive fibrosing ILD other than IPF (TOMORROW, INPULSIS, SENSCIS, INBUILD). *AST* aspartate transaminase, *ALT* alanine transaminase, *BID* twice daily, *CI* confidence interval, *C*_*pre,ss*_ pre-dose drug concentration in plasma at steady state, *ILD* interstitial lung disease, *IPF* idiopathic pulmonary fibrosis, *SSc-ILD* systemic sclerosis-associated interstitial lung disease

Figure 1 shows the association between nintedanib exposure and the probability of a liver enzyme elevation stratified by covariates of gender and study, based on the final exposure–liver enzyme elevation model using data from all studies (TOMORROW, INPULSIS, SENSCIS and INBUILD). In addition, the figure shows this relationship for the model from the sensitivity analysis using the 2019 updated reference ranges for ALT and AST (see “Materials and methods” section).

### Exposure–diarrhea analyses

Exposure–diarrhea analyses based on IPF studies indicated that the proportion of patients experiencing diarrhea of any intensity increased with a higher nintedanib dose. However, there was no clear association between the risk of diarrhea and exposure within each dose group. As such, the initially developed exposure–diarrhea model (consisting of a Weibull baseline hazard and a sigmoidal E_max_ drug effect function; see Additional file 1: Table S6) was inferior to the dose-group models (using actual or intention-to-treat dose as predictor of the diarrhea risk; see Additional file 1: Table S7) in terms of objective function values. This was supported by visual predictive checks, where a good predictive performance across treatment groups was observed for the dose-group model (Additional file 1: Figure S2a). The exposure–diarrhea model, however, showed over- and under-prediction of diarrhea risk for the low- and high-exposure groups, respectively (Additional file 1: Figure S2b).

The lack of exposure-dependency of diarrhea was also apparent during covariate assessment using both the dose-group models and the exposure–diarrhea models. The outcomes of covariate assessments were consistent between the dose-group and exposure–diarrhea models, except for the effect of Asian ethnicity and smoking. These covariates had no effect (for smoking) or a weaker effect (for Asian subpopulations) on diarrhea risk in the dose-group models as compared to the exposure–diarrhea models. Both observed and predicted C_pre,ss_ models showed that Asian patients and those who had never smoked had a lower risk of developing diarrhea compared with White patients and current or ex-smokers, respectively (Additional file 1: Table S8). However, given that Asian patients and former/non-smokers were found to have a higher exposure compared with White patients and current smokers in a PopPK analysis [10], the decreased diarrhea risk in Asian patients and non-smokers may compensate for the difference in exposure and thus result in overall comparable risk for a given dose.

The exploratory analyses matching patients in the 150 and 100 mg BID treatment groups of the IPF studies by exposure further supported the exposure–diarrhea assessment. A good congruency of plasma exposure between the 150 mg BID and the 100 mg BID treatment groups was achieved by optimal matching (median observed C_pre,ss_ of 4.55 ng/mL and 4.60 ng/mL in the 100 mg BID and the 150 mg BID treatment group, respectively, and corresponding median predicted C_pre,ss_ values of 5.56 ng/mL and 6.93 ng/mL, respectively; Additional file 1: Table S9). Hence, the median C_pre,ss_ in the optimally matched 150 mg BID group had significantly lower exposure compared with the overall 150 mg BID treatment group (observed and predicted C_pre,ss_ of 9.7 ng/mL and 10.5 ng/mL, respectively). Nevertheless, a similar incidence of diarrhea in the optimally matched 150 mg BID treatment group as in the overall 150 mg BID treatment groups without optimal matching from IPF trials was observed (~ 60%; Tables 3 and 5). Likewise, despite similar nintedanib exposure (Additional file 1: Table S9), a significantly lower incidence of diarrhea was observed in the 100 mg BID treatment group compared with the optimally matched 150 mg BID group.

**Table 5:** Incidence of diarrhea in the 100 mg BID treatment group and the 150 mg BID treatment group, optimally matched by nintedanib plasma exposure (observed and predicted C_pre,ss_ at starting dose level) in IPF trials (TOMORROW, INPULSIS-1 and INPULSIS-2)

100 mg BID				150 mg BID (optimally matched by predicted C_pre,ss_)			
No diarrhea		Diarrhea event		No diarrhea		Diarrhea event	
N	%	N	%	N	%	N	%
54	62.8	32	37.2	65	37.8	107	62.2

100 mg BID				150 mg BID (optimally matched by observed C_pre,ss_)			
No diarrhea		Diarrhea event		No diarrhea		Diarrhea event	
N	%	N	%	N	%	N	%
50	62.5	30	37.5	66	41.3	94	58.8

*BID* twice daily, *C*_*pre,ss*_ pre-dose drug concentration in plasma at steady state, *N* Number of patients

Consistent with the findings in IPF, exploratory analyses with data from the SENSCIS trial and the INBUILD trial indicated that the exposure dependency of diarrhea risk in patients in the 150 mg nintedanib BID treatment group was either non-existent or very limited. A slight (if any) increase in the frequency of mild diarrhea events was found in patients belonging to the group in the highest exposure tertile in the SENSCIS and INBUILD trials. For moderate or severe diarrhea events, no exposure dependency was detected across all trials (Additional file 1: Table S10).

## Discussion

The exposure–safety analyses reported here were conducted to understand the relationship between nintedanib exposure and safety in terms of liver enzyme elevations and diarrhea, and support dose selection for patients with IPF, other chronic fibrosing ILDs with a progressive phenotype and for SSc-ILD. Data from several BID doses in IPF trials (50–150 mg) provided a relatively wide range of nintedanib exposure, enabling a good exploration of the exposure–safety relationship. Additional data from patients with SSc-ILD and progressive fibrosing ILDs other than IPF from the SENSCIS and INBUILD trials allowed comprehensive analyses across indications.

As the assessment of liver enzyme elevation events in IPF was based on a limited number of events (~ 1% in the placebo group and ~ 5% in the 150 mg nintedanib BID group) and no difference between patient populations was to be expected (i.e. the mechanism was considered indication-independent), safety data on liver enzyme elevations from studies in IPF were combined with data from studies in SSc-ILD and progressive fibrosing ILDs other than IPF (SENSCIS and INBUILD) for analysis. As such, a higher power for the detection of potential covariate effects was obtained than by using data from single studies only.

Both observed and PopPK model-predicted C_pre,ss_ values were selected as exposure measures for the analyses in IPF. For the scaling of observed C_pre,ss_, the starting dose was used. The predicted C_pre,ss_ values were implemented time-dependently thus also taking into account dose reductions and treatment interruptions during trials. The two exposure variables were highly correlated, as both were derived using actual plasma concentration measurements from the trials. However, for the model-predicted values, patient demographics, pharmacokinetic variability and actual dosing history was additionally considered. This was assumed to further minimize bias, as it enabled derivation of exposure variables also for patients without a measured nintedanib plasma concentration and dose changes during the trial were taken into account. As analyses in IPF suggested consistent results for the two exposure measures, analyses in SSc-ILD and progressive fibrosing ILDs other than IPF were performed by using time-matched predicted C_pre,ss_ values only. Of note, the relationship between exposure and safety risks tended to be steeper when using predicted C_pre,ss_ than by using observed C_pre,ss_. Hence, use of predicted C_pre,ss_ led to larger changes in the safety risks for subgroups with altered nintedanib exposure being predicted than through use of observed C_pre,ss_ (conservative approach).

With respect to liver enzyme elevations, a positive correlation between nintedanib plasma exposure and ALT or AST elevations ≥ 3 × ULN was found based on initial analyses in patients with IPF (using combined data from the TOMORROW and INPULSIS trials). This was confirmed by analyzing combined data from trials in IPF, SSc-ILD (SENSCIS) and chronic fibrosing ILDs with a progressive phenotype other than IPF (INBUILD). On top of the exposure-related risk (covering known factors leading to exposure increase such as Asian race, low body weight or high age), females were estimated on average to have a 3.7-fold higher risk of experiencing ALT or AST elevations ≥ 3 × ULN than males, and data in SSc-ILD and chronic fibrosing ILDs with a progressive phenotype other than IPF were again in line with findings from initial analyses in IPF.

During covariate assessment, no difference in the exposure–liver enzyme elevation relationship was found between patients included in the IPF trials and patients with SSc-ILD from the SENSCIS trial. Likewise, no clear difference of this relationship between patients from the INBUILD trial as compared to IPF trials or the SENSCIS trial was detected. Although the main analysis, using reference ranges for ALT and AST defined in 2014 by the central laboratory, suggested an approximately twofold higher (exposure- and gender-adjusted) probability of transaminase elevations for patients in the INBUILD trial than for patients in the IPF or SENSCIS trials, no significant difference between trials was present in the sensitivity analysis using 2019 updated reference ranges for ALT and AST. These reference ranges were established by the central laboratory provider independently from the INBUILD trial in 2019 (see “Materials and methods” section) and were more closely aligned to those used in previous nintedanib studies (see Additional file 1: Table S1). Overall, it needs to be taken into account that due to the lack of standardized reference ranges for ALT and AST measurements, differences between laboratories can affect the comparability of assessments on drug-induced liver disease based on ALT or AST [24–26]. Characteristics of the local reference population used for the determination of reference ranges or differences in the methodology by manufacturers to establish recommended reference intervals have been identified as relevant factors contributing to this variability [27, 28]. With this in mind, the 2019 updated reference ranges for the INBUILD trial (more closely aligned to the reference ranges from previous nintedanib trials) might be more appropriate for comparison of liver enzyme elevation events between the different nintedanib studies than the 2014 reference ranges used for the INBUILD primary analysis. The 2014 reference ranges for INBUILD (with lower ULNs than in previous nintedanib trials) are considered to provide a more conservative estimate of the incidence of liver enzyme elevation events. As such, the use of these values led to a higher number of observed events (by a factor of ~ 2) in the INBUILD trial than in IPF trials or in the SENSCIS trial, and trigger a study effect in the exposure–liver enzyme elevation model. However, the sensitivity analysis indicates that numerical differences in liver enzyme elevations between trials can be explained by assay differences, data variability and patient demographics (e.g. distribution of females or factors influencing exposure such as low/high body weight, age or Asian ethnicity) such that a clear population effect cannot be determined. The relationship between nintedanib plasma exposure and ALT or AST elevations ≥ 3 × ULN was weak to moderate across all models and indications (taking into account the steepness of the exposure–safety curve), and was therefore comparable between studies in IPF, SSc-ILD and chronic fibrosing ILDs with a progressive phenotype other than IPF. Liver enzyme elevations normalized in the majority of patients in nintedanib trials either spontaneously or with dose reduction, treatment interruption or discontinuation.

The comprehensive analyses with regard to diarrhea presented here indicate that there is no association between exposure and the risk of diarrhea based on data from IPF, SSc-ILD or chronic fibrosing ILDs with a progressive phenotype other than IPF. However, a clear relationship between the dose administered and diarrhea was observed. This suggests that local gut concentrations might be more relevant than plasma exposure for the occurrence of diarrhea.

The exposure–safety analyses described here, in combination with recently published exposure–efficacy analyses for nintedanib [29] support the therapeutic dose of 150 mg nintedanib BID in patients with chronic progressive fibrosing ILDs overall and in subgroups of patients, where nintedanib plasma exposure may be altered (e.g. due to known factors leading to an exposure change such as Asian race, low or high body weight and low or high age). Previous PopPK analyses indicated that intrinsic or extrinsic factors have only a small to moderate influence on nintedanib plasma exposure [10]. Thus, single covariate effects of Asian race, body weight or age did not change the plasma exposure by more than 50% and were well within the variability range of nintedanib. In addition, recently published exposure-efficacy analyses [29] indicate that an increase in exposure might still be beneficial in terms of efficacy. At the same time, for an exposure increase by up to 50%, a limited safety impact is expected, as based on the current analysis, plasma exposure has no influence on diarrhea occurrence and only a moderate influence on transaminase elevations (Fig. 1). Adverse events were manageable by dose reductions and treatment interruptions. Based on this, altered nintedanib exposure does not warrant a priori dose adjustment (excluding patients with hepatic impairment having specific labelling recommendations [1, 2]). Due to a potentially higher frequency of liver enzyme elevations, patients with elevated nintedanib exposure (e.g. due to Asian race, low body weight, high age or combinations of these risk factors) should, however, be closely monitored for tolerability. Additional assessments on tolerability and safety and the appropriate use of nintedanib have been published previously [30].

## Conclusions

In summary, results of exposure–safety analyses for nintedanib were consistent across nintedanib studies including patients with IPF, other chronic fibrosing ILDs with a progressive phenotype and SSc-ILD. A positive correlation between nintedanib exposure and ALT or AST elevations in general and female gender as an exposure-independent risk factor was found. This relationship was considered weak to moderate across different indications.

With regard to diarrhea, the actual dose administered was found to be a better predictor of the risk of experiencing diarrhea than plasma exposure, suggesting that local gut concentrations may be more relevant than plasma exposure. Therefore, a change in diarrhea risk is not expected for patients with altered nintedanib exposure (e.g. due to low/high age, body weight or Asian race). Based on this, no a priori dose adjustment is recommended in patients with altered nintedanib exposure (except for patients with hepatic impairment substantially affecting nintedanib plasma exposure). However, due to a potentially higher frequency of AEs, close monitoring for tolerability is warranted for patients with elevated nintedanib exposure (e.g. due to Asian race, low body weight, high age or combinations of these risk factors).

The presented exposure–safety analyses, in combination with recently published exposure–efficacy analyses for nintedanib [29], provide a platform to assess the risk–benefit profile of nintedanib in IPF, other chronic fibrosing ILDs with a progressive phenotype and SSc-ILD, and support the therapeutic dose of 150 mg nintedanib BID across different indications of chronic fibrosing ILDs.

## Supplementary Information

**Additional file 1.** Supplementary tables and figures.

## Data Availability

The datasets used and/or analysed during the current study can be made available from the corresponding author on reasonable request.

## Abbreviations

AE: Adverse event
ALT: Alanine transaminase
AST: Aspartate transaminase
BID: Twice daily
CI: Confidence interval
C_pre,ss_: Pre-dose drug concentrations at steady state
E_max_: Maximum effect
ILD: Interstitial lung disease
IPF: Idiopathic pulmonary fibrosis
PopPK: Population pharmacokinetic
SSc: Systemic sclerosis
ULN: Upper limit of normal
