# Supplementary material for: Exposure–safety analyses of nintedanib in patients with chronic fibrosing interstitial lung disease
Source: BMC Pulm Med. 2021 Jul 21;21:244. doi: 10.1186/s12890-021-01598-0 (PMC8293560; doi:10.1186/s12890-021-01598-0)
Supplement: Supplementary file 1 — Additional file 1. Supplementary tables and figures. [file 12890_2021_1598_MOESM1_ESM.docx]

**SUPPLEMENTARY MATERIAL**

**Supplementary Figure S1.** Visual predictive check showing the Kaplan–Meier curve of time to first liver enzyme elevation based on pooled data from IPF (TOMORROW, INPULSIS), SSc-ILD (SENSCIS) and progressive fibrosing ILD other than IPF (INBUILD): (A) overall; (B) by gender; (C) by study; and (D) by exposure (based on C_pre,ss_)

**(A)**

**(B)**
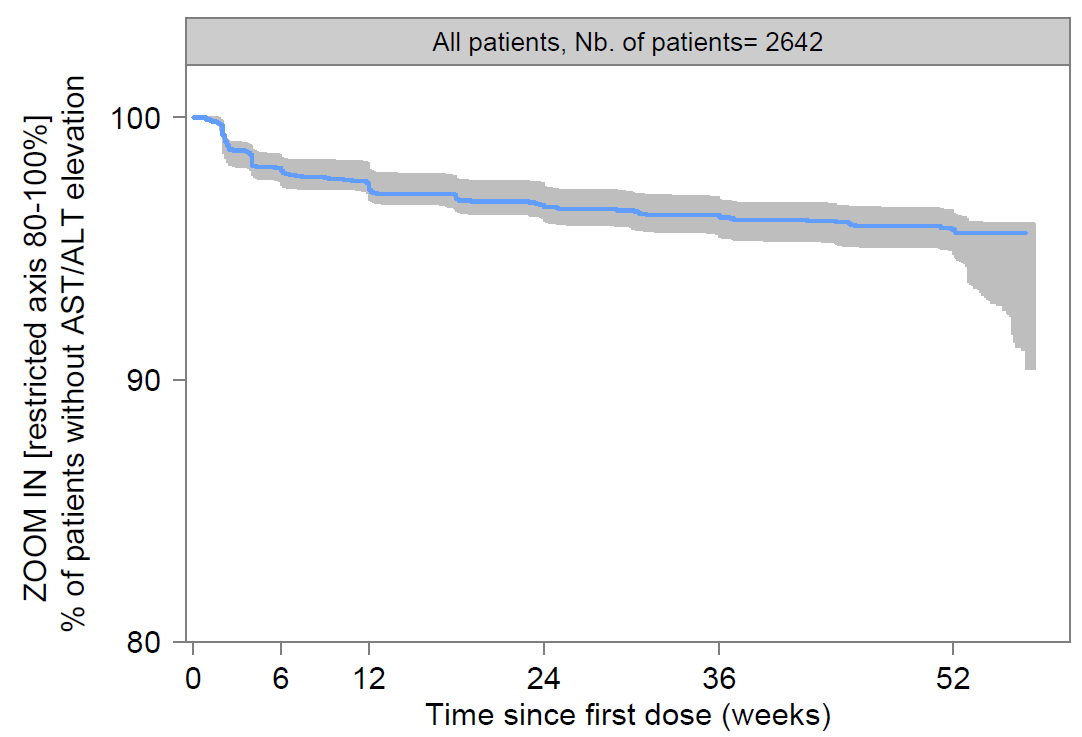

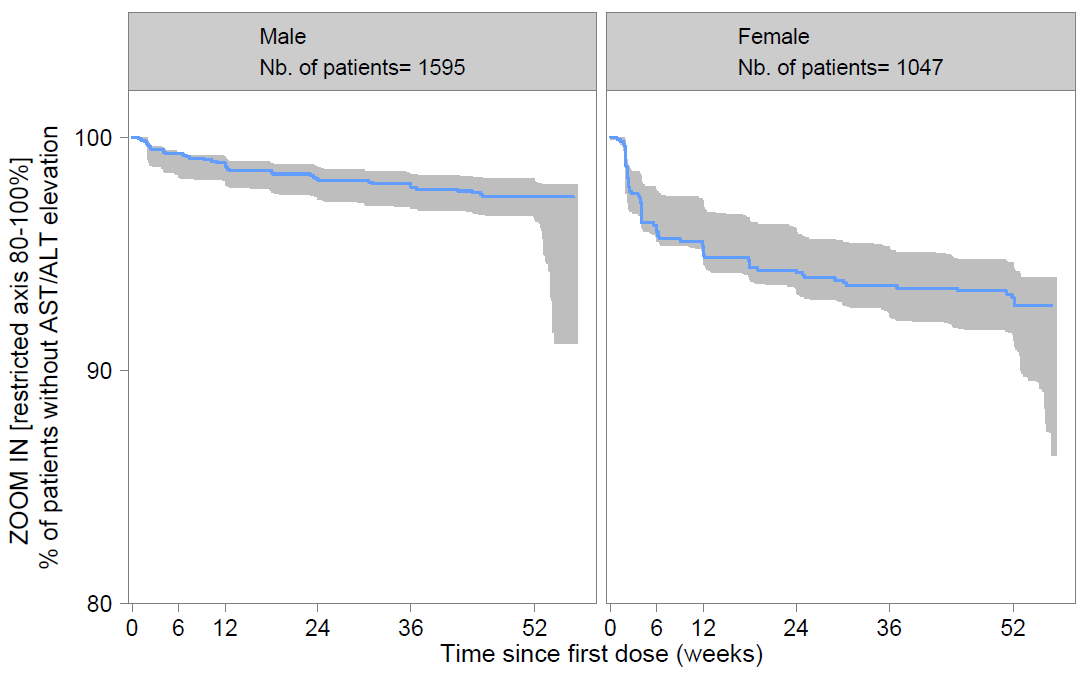


**(C)**


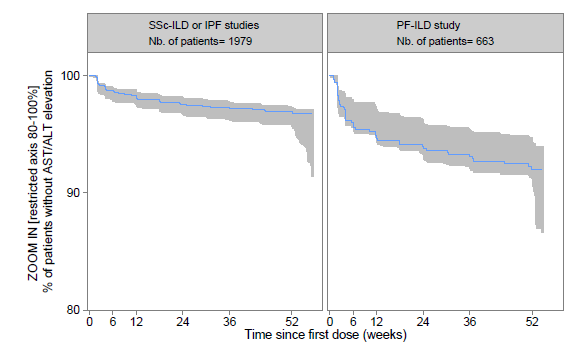


**(D)**


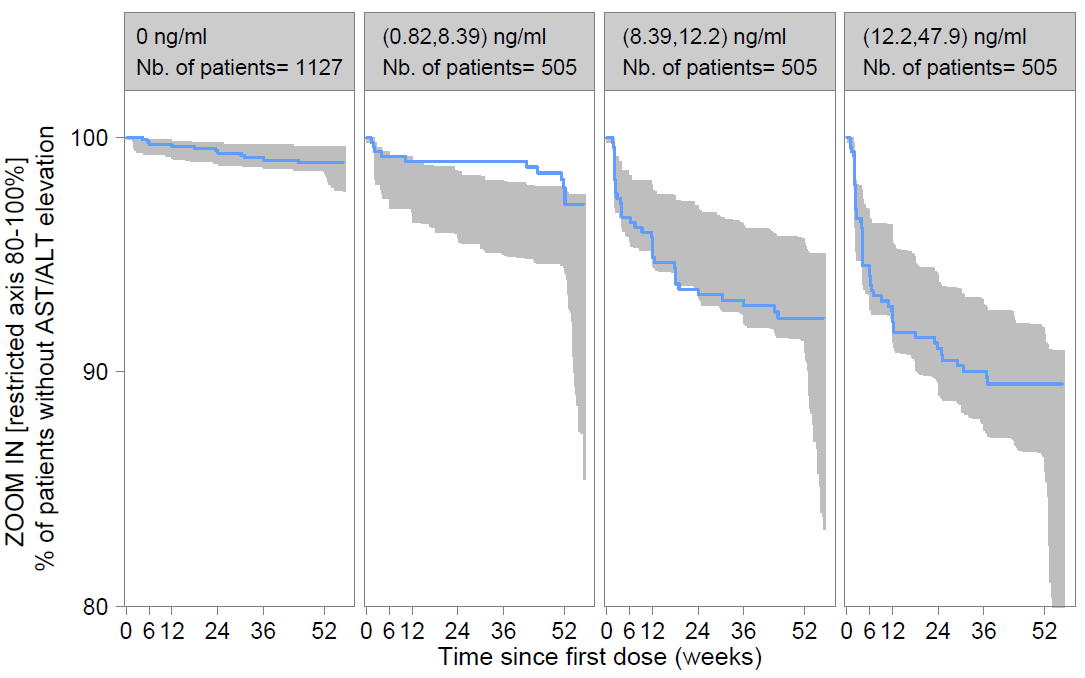


Abbreviations: ALT, alanine transaminase; AST, aspartate transaminase; IPF, idiopathic pulmonary fibrosis; PF-ILD, progressive fibrosing interstitial lung disease; SSc-ILD, systemic sclerosis-associated interstitial lung disease; C_pre,ss_, pre-dose drug concentration in plasma at steady state.

**Supplementary Figure S2.** Visual predictive checks by treatment group for the diarrhea models using (A) treatment group (ITT) and (B) exposure (observed C_pre,ss_ levels) as predictor of diarrhea.

**(A)** Treatment group (ITT) based model


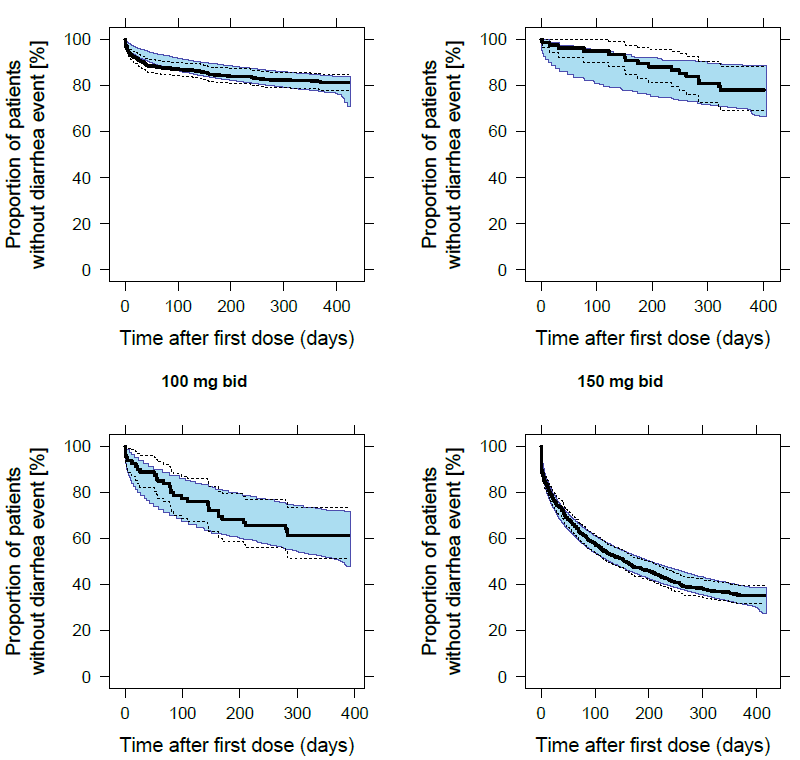


**(B)** Exposure based model


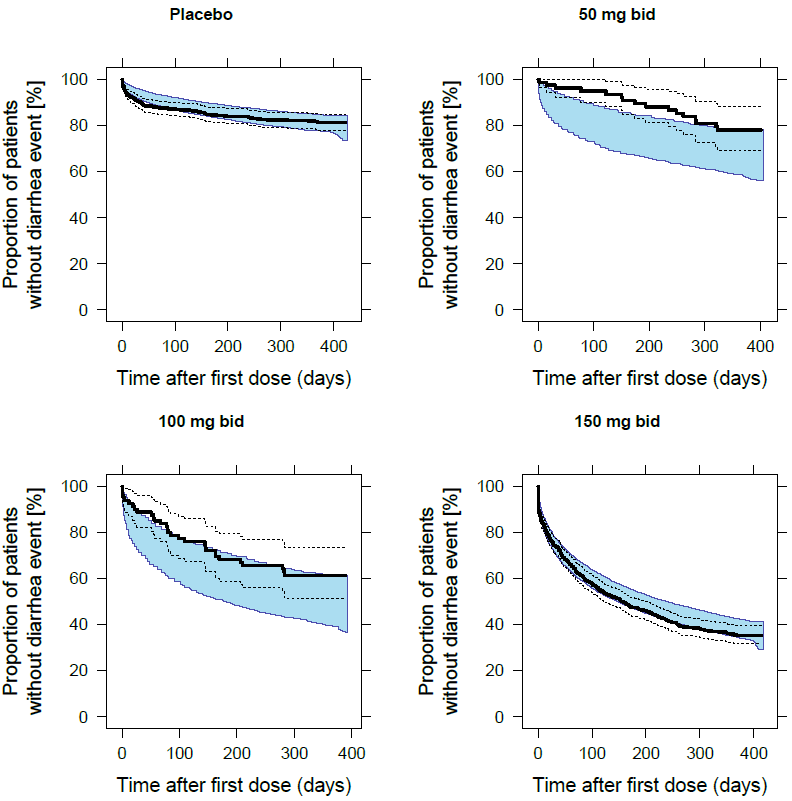


Kaplan–Meier plots show the proportion of patients without diarrhea events versus time after first dose (days). Blue shaded area represents the 95% confidence interval of simulated data (1000 simulations) based on the two models. Continuous and broken lines correspond to real data and standard errors, respectively.

Abbreviations: BID, twice daily; C_pre,ss_, pre-dose drug concentration in plasma at steady state.

**Supplementary Table S1.** Reference ranges for ALT and AST in INPULSIS/SENSCIS and in INBUILD

| **Marker** | **Study** | **Sex** | **Age (Y)** | **Range (U/L)** |
| --- | --- | --- | --- | --- |
| ALT | INPULSIS SENSCIS | F/M | 18+ | 0–48 |
|  | INBUILD (2014 reference range used for primary analysis) | F | 18–69 | 6–34 |
|  |  |  | 69–150 | 6–32 |
|  |  | M | 18–69 | 6–43 |
|  |  |  | 69–150 | 6–35 |
|  | INBUILD (2019 updated reference range used for sensitivity analysis) | *F* | *18+* | *4–43* |
|  |  | *M* | *18+* | *5–48* |
| **Marker** | **Study** | **Sex** | **Age (Y)** | **Range (U/L)** |
| AST | INPULSIS/SENSCIS | F/M | 18–64 | 0–42 |
|  |  | F/M | 65+ | 0–55 |
|  | INBUILD (2014 reference range used for primary analysis) | F | 18–150 | 9–34 |
|  |  | M | 18–150 | 11–36 |
|  | INBUILD (2019 updated reference range used for sensitivity analysis) | *F/M* | *18+* | *8–40* |

Abbreviations: ALT, alanine transaminase; AST, aspartate transaminase; F, female; M, male; U/L, units per litre; Y, year.

**Supplementary Table S2.** Parameter estimates for the initial exposure–liver enzyme elevation model in IPF (using observed and predicted C_pre,ss_ levels)

| **Parameter** | **Unit** | **Estimate (%RSE)** | |
| --- | --- | --- | --- |
|  |  | **Model based on observed C_pre,ss_** | **Model based on predicted C_pre,ss_** |
| Scale factor λ (Weibull distribution) | 1/day | 0.000542 (64.5) | 0.000477 (67.3) |
| Shape factor $\gamma$ (Weibull distribution) |  | 0.458 (18.9) | 0.410 (17.8) |
| Log-linear coefficient of drug effect_male_ |  | 0.579 (31.0) | 0.732 (30.7) |
| Gender on log-linear coefficient_female_ |  | 0.691 (55.7) | 0.758 (45.1) |

**Description of models:**

**Model based on observed C_pre,ss_:** 1283 subjects, 1283 observations, objective function of 432.83

**Model based on predicted C_pre,ss_:** 1403 subjects, 1403 observations, objective function of 493.86

$$h\left( t \right)=\lambda*f\left( Cpre,ss \right)*\gamma*t^{\gamma-1}$$

$$f\left( Cpre,ss \right)= e^{PH\_Cpre*\log\left( 1+Cpre,ss \right)*(1+PH_{Gender})}$$

Abbreviations: λ, scale parameter (Weibull distribution); γ, shape parameter (Weibull distribution) ; C_pre,ss_, observed or predicted pre-dose drug concentration in plasma at steady state; h(t), hazard at time t; IPF, idiopathic pulmonary fibrosis; PH_C_pre_, log-linear coefficient of the drug effect; PH_Gender_, gender effect on log-linear coefficient referring to females (for males this parameter is set to 0); RSE, relative standard error.

**Supplementary Table S3.** Tested covariates and results from covariate analysis for the exposure–liver enzyme elevation relationship in IPF based on observed and predicted C_pre,ss_

|  | **Significant relationships**^#^ | |
| --- | --- | --- |
| **Covariate** | **Model based on observed C_pre,ss_** | **Model based on predicted C_pre,ss_** |
| Age | - | - |
| Height | - | - |
| Body weight | - | - |
| Body surface area | - | - |
| Gender (male vs. female) | - | ✓ (dOFV=18.2) |
| Smoking status (never vs. ex- vs. current) | - | - |
| Asian subpopulations* | - | - |
| Study (TOMORROW vs. INPULSIS-1 vs. INPULSIS-2) | - | - |

^#^Covariates were tested on the coefficient of the log-linear relationship (drug effect parameter).

✓: significant parameter–covariate relationship after backward elimination step. - : no statistically significant parameter-covariate relationship after backward elimination step.

* The recorded ethnicity for Asian subpopulations was based on the recorded race of the patient and the country in which a study site was located. Subgroups: White (including Black) vs. Chinese vs. Taiwanese vs. Indian vs. Japanese vs. Korean vs. other Asian (patients with Asian race not assigned to one of the above Asian subgroups or American Indian/Alaska native). Patients with missing race were grouped with White patients (as race information was not collected for patients with study site located in France due to local laws).

Abbreviations: C_pre,ss_, pre-dose drug concentration in plasma at steady state; dOFV, difference in objective function value; IPF, idiopathic pulmonary fibrosis.

**Supplementary Table S4.** Tested covariates and results from covariate analysis for the exposure–liver enzyme elevation relationship based on combined IPF/SSc-ILD data

| **Covariate** | **Significant relationships**^#^ **Model based on predicted C_pre,ss_** |
| --- | --- |
| Age | - |
| Body weight | - |
| Gender (male vs. female) | ✓ (dOFV=18.2) |
| Race* | - |
| Asian subpopulations** | - |
| ATA status (positive vs. negative) | - |
| Mycophenolate (mofetil/sodium/acid) use at baseline (yes vs. no) | - |
| SSc subtype (diffuse cutaneous SSc vs. limited cutaneous SSc) | - |
| Study (TOMORROW/INPULSIS combined vs. SENSCIS) | - |

^#^Covariates were tested on the coefficient of the log-linear relationship (drug effect parameter).

✓: significant parameter–covariate relationship after backward elimination step. - : no statistically significant parameter-covariate relationship after backward elimination step.

*White (including Multiple) vs. Black vs. Asian (including American Indian/Alaska native and Hawaiian/Pacific Islander). Patients with missing race were grouped with White patients (as race information was not collected for patients with study site located in France due to local laws).

** The recorded ethnicity for Asian subpopulations was based on the recorded race of the patient and the country in which a study site was located. Subgroups: White with study site located in Europe vs. White with study site located in Asia vs. White with study site located in North America vs. White with study site located in Rest of the World vs. Chinese (including Taiwanese) vs. Korean vs. Indian vs. Japanese vs. other Asian (patients with Asian race not assigned to one of the above Asian subgroups or American Indian/Alaska native or Hawaiian/Pacific Islander) vs. Black.

Abbreviations: ATA, anti-topoisomerase I antibody; C_pre,ss_, pre-dose drug concentration in plasma at steady state; dOFV, difference in objective function value; IPF, idiopathic pulmonary fibrosis; SSc-ILD, systemic sclerosis-associated interstitial lung disease.

**Supplementary Table S5.** Tested covariates and results from covariate analysis for the exposure–liver enzyme elevation relationship based on combined IPF/SSc-ILD/ progressive fibrosing ILD data

| **Covariate** | **Significant relationships**^#^ **Model based on predicted C_pre,ss_** |
| --- | --- |
| Age | - |
| Body weight | - |
| Gender (male vs. female) | ✓ (dOFV=28.8) |
| Race* | - |
| Asian subpopulations** | - |
| Methotrexate use at baseline (yes vs. no) | - |
| DMARDs with known hepatotoxic effects use at baseline (yes vs. no) | - |
| Disease severity based on baseline FVC % predicted | - |
| Study (TOMORROW/INPULSIS/SENSCIS combined vs. INBUILD) | ✓ (dOFV=12.1) |

^#^Covariates were tested on the coefficient of the log-linear relationship (drug effect parameter).

✓: significant parameter–covariate relationship after backward elimination step. - : no statistically significant parameter-covariate relationship after backward elimination step.

*White (including Multiple) vs. Black vs. Asian (including American Indian/Alaska native and Hawaiian/Pacific Islander). Patients with missing race were grouped with White patients (as race information was not collected for patients with study site located in France due to local laws).

** The recorded ethnicity for Asian subpopulations was based on the recorded race of the patient and the country in which a study site was located. Subgroups: White vs. Chinese (including Taiwanese) vs. Indian vs. Japanese vs. Korean vs. other Asian (patients with Asian race not assigned to one of the above Asian subgroups or American Indian/Alaska native or Hawaiian/Pacific Islander) vs. Black.

Abbreviations: DMARD, disease-modifying antirheumatic drug; dOFV, objective function value; FVC, forced vital capacity; ILD, interstitial lung disease; IPF, idiopathic pulmonary fibrosis; SSc-ILD, systemic sclerosis-associated interstitial lung disease.

**Supplementary Table S6.** Parameter estimates from the initial diarrhea models in IPF before covariate analysis using exposure (observed or predicted C_pre,ss_) as predictors of diarrhea risk

| **Parameter** | **Unit** | **Estimate (%RSE)** | | |
| --- | --- | --- | --- | --- |
|  |  | **Model based on observed C_pre,ss_** | | **Model based on predicted C_pre,ss_** |
| Scale factor $\lambda$ (Weibull distribution) | 1/day | 0.0124 (15.0) | 0.0119 (14.4) | |
| Shape factor $\gamma$ (Weibull distribution) | **-** | 0.477 (4.00) | 0.486 (3.75) | |
| E_max_ | **-** | 4.15 (16.1) | 4.10 (15.4) | |
| EC_50_ | ng/mL | 3.65 (13.5) | 4.92 (8.72) | |
| Hill | - | 3.30 (36.6) | 6.57 (62.8) | |

**Description of models:**

**Model based on observed C_pre,ss_:** 1283 subjects, 1283 observations, objective function of 6963.73

**Model based on predicted C_pre,ss_:** 1403 subjects, 1403 observations, objective function of 7678.26

$$h\left( t \right)=\lambda*f\left( Cpre,ss \right)*\gamma*t^{\gamma-1}$$

$$f\left( Cpre,ss \right)=1+\frac{E_{max}*{Cpre,ss}^{Hill}}{{{EC}_{50}}^{Hill}+{Cpre,ss}^{Hill}}$$

Abbreviations: λ, scale parameter (Weibull distribution); γ, shape parameter (Weibull distribution); BID, twice daily; C_pre,ss_, pre-dose drug concentration in plasma at steady state; EC_50_, half maximum-effect concentration (sigmoidal maximum drug effect function); E_max_, maximum effect (sigmoidal maximum drug effect function); h(t), hazard at time t; Hill, Hill coefficient (sigmoidal maximum drug effect function); IPF, idiopathic pulmonary fibrosis; RSE, relative standard error.

**Supplementary Table S7.** Parameter estimates from the initial diarrhea models in IPF before covariate analysis using dose group (actual and ITT) as predictors of diarrhea risk

| **Parameter** | **Unit** | **Estimate (%RSE)** | |
| --- | --- | --- | --- |
|  |  | **Model based on treatment group (ITT)** | **Model based on dose group (actual)** |
| Scale factor $\lambda$ (Weibull distribution) |  |  |  |
| Placebo | 1/day | 0.0125 (15.2) | 0.0117 (14.8) |
| 50 mg BID | 1/day | 0.0136 (27.3) | 0.0125 (26.4) |
| 100 mg BID | 1/day | 0.0288 (21.1) | 0.0282 (19.2) |
| 150 mg BID | 1/day | 0.0609 (11.3) | 0.0603 (10.6) |
| Shape factor $\gamma$ (Weibull distribution) | - | 0.478 (3.99) | 0.490 (3.74) |

**Description of models:**

**Model with treatment group (ITT) as predictor (same population as for model based on observed C_pre,ss_):** 1283 subjects, 1283 observations, objective function of 6951.79

**Model with actual dose group as predictor (same population as for model based on predicted C_pre,ss_):** 1403 subjects, 1403 observations, objective function of 7668.20

Model allowed for different λs for the different dose groups

$$h\left( t \right)=\lambda*\gamma*t^{\gamma-1}$$

Abbreviations: λ, scale parameter (Weibull distribution); γ, shape parameter (Weibull distribution); BID, twice daily; h(t), hazard at time t; IPF, idiopathic pulmonary fibrosis; ITT, intention to treat; RSE, relative standard error.

**Supplementary Table S8.** Tested covariates and results from covariate analysis for exposure–diarrhea relationships and dose–diarrhea relationships of nintedanib in IPF

|  | **Significant relationships**^#^ | | | |
| --- | --- | --- | --- | --- |
| **Covariate** | **Model based on observed C_pre,ss_** | **Model based on predicted C_pre,ss_** | **Model based on dose group (ITT)** | **Model based on dose group (actual)** |
| Age | - | - | - | - |
| Height | - | - | - | - |
| Body weight | - | - | - | - |
| Body surface area | - | - | - | - |
| Gender (male vs. female) | - | - | - | - |
| Smoking status (never vs. ex- vs. current) | ✓ (on E_max_; dOFV=12.0) | ✓ (on E_max_; dOFV=12.2) | - | - |
| Asian subpopulations* | ✓ (on EC_50_;  dOFV=34.5) | ✓ (on EC_50_;  dOFV=34.1) | ✓ (on λ; dOFV=25.6) | ✓ (on λ; dOFV=24.9) |
| Study (TOMORROW vs. INPULSIS-1 vs. INPULSIS-2) | - | - | - | - |

^#^Covariates were tested on parameters λ (for all models) and EC_50_, E_max_ and Hill (for exposure-response models only).

✓: significant parameter–covariate relationship after backward elimination step. - : no statistically significant parameter-covariate relationship after backward elimination step.

* The recorded ethnicity for Asian subpopulations was based on the recorded race of the patient and the country in which a study site was located. Subgroups: White (including Black) vs. Chinese vs. Taiwanese vs. Indian vs. Japanese vs. Korean vs. other Asian (patients with Asian race not assigned to one of the above Asian subgroups or American Indian/Alaska native). Patients with missing race were grouped with White patients (as race information was not collected for patients with study site located in France due to local laws).

Abbreviations: λ, scale parameter of the hazard function (Weibull distribution); C_pre,ss_, pre-dose drug concentration in plasma at steady state; dOFV, difference in objective function value; EC_50_, half maximum-effect concentration (sigmoidal maximum drug effect function); E_max_, maximum effect (sigmoidal maximum drug effect function); Hill, Hill coefficient (sigmoidal maximum drug effect function); IPF, idiopathic pulmonary fibrosis; ITT, intention to treat.

**Supplementary Table S9.** Descriptive statistics of observed and predicted C_pre,ss_ at starting dose level in 100 mg and optimally matched 150 mg treatment groups (1:2 matching) from IPF trials (TOMORROW, INPULSIS-1 and INPULSIS-2)

| **Observed C_pre,ss_ (ng/mL)** | | | | | | | | | | | | | | | |
| --- | --- | --- | --- | --- | --- | --- | --- | --- | --- | --- | --- | --- | --- | --- | --- |
| **100 mg BID treatment group** | | | | | | | | | | | | | | | |
| N | Min | | P5 | | P25 | | Median | | P75 | | P95 | | Max | | Mean |
| 80 | 0.75 | | 1.68 | | 3.09 | | 4.55 | | 6.58 | | 11.71 | | 13.63 | | 5.21 |
| **Optimally matched 150 mg BID treatment group** | | | | | | | | | | | | | | | |
| N | | Min | | P5 | | P25 | | Median | | P75 | | P95 | | Max | Mean |
| 160 | | 1.75 | | 2.38 | | 3.69 | | 4.60 | | 6.58 | | 11.71 | | 13.66 | 5.48 |
| **Predicted C_pre,ss,_ (ng/mL)** | | | | | | | | | | | | | | | |
| **100 mg BID treatment group** | | | | | | | | | | | | | | | |
| N | Min | | P5 | | P25 | | Median | | P75 | | P95 | | Max | | Mean |
| 86 | 1.59 | | 2.48 | | 3.97 | | 5.56 | | 7.14 | | 11.61 | | 14.16 | | 6.01 |
| **Optimally matched 150 mg BID treatment group** | | | | | | | | | | | | | | | |
| N | | Min | | P5 | | P25 | | Median | | P75 | | P95 | | Max | Mean |
| 172 | | 3.22 | | 4.66 | | 5.97 | | 6.93 | | 7.56 | | 11.61 | | 14.16 | 7.15 |

Abbreviations: BID, twice daily; C_pre,ss_, pre-dose drug concentration in plasma at steady state; max, maximum; min, minimum; N, number of subjects; Px, xth percentile.

**Supplementary Table S10.** Incidence of diarrhea over 52 weeks in the TOMORROW, INPULSIS, SENSCIS and INBUILD studies, stratified by predicted exposure tertiles and severity grade

| **Predicted C_pre,ss_ at 150 mg BID [ng/mL]** | **Patients, n (%)** | | | | |
| --- | --- | --- | --- | --- | --- |
|  | **Overall** | **None** | **Mild** | **Moderate** | **Severe** |
| **TOMORROW and INPULSIS-1/2 (IPF)** | | | | | |
| 0 (placebo) | 508 (100) | 417 (82) | 72 (14) | 17 (3) | 2 (<1) |
| Low: ≤9.2 | 241 (100) | 92 (38) | 73 (30) | 65 (27) | 11 (5) |
| Intermediate: [9.2; 12.4] | 241 (100) | 100 (41) | 76 (32) | 58 (24) | 7 (3) |
| High: >12.4 | 241 (100) | 86 (36) | 99 (41) | 49 (20) | 7 (3) |
| **SENSCIS (SSc-ILD)** | | | | | |
| 0 (placebo) | 288 (100) | 197 (68) | 61 (21) | 27 (9) | 3 (1) |
| Low: ≤8.34 | 96 (100) | 27 (28) | 33 (34) | 32 (33) | 4 (4) |
| Intermediate: [8.34; 11.5] | 96 (100) | 24 (25) | 28 (29) | 39 (41) | 5 (5) |
| High: >11.5 | 96 (100) | 19 (20) | 47 (49) | 27 (28) | 3 (3) |
| **INBUILD (chronic fibrosing ILDs with a progressive phenotype other than IPF)** | | | | | |
| 0 (placebo) | 331 (100) | 252 (76) | 59 (18) | 17 (5) | 3 (1) |
| Low: ≤10.2 | 111 (100) | 41 (37) | 44 (40) | 23 (21) | 3 (3) |
| Intermediate: [10.2; 14.0] | 110 (100) | 37 (34) | 48 (44) | 22 (20) | 3 (3) |
| High: >14.0 | 111 (100) | 33 (30) | 55 (50) | 20 (18) | 3 (3) |

Abbreviations: BID, twice daily; C_pre,ss_, pre-dose drug concentration in plasma at steady state; ILD, interstitial lung disease; IPF, idiopathic pulmonary fibrosis; SSc, systemic sclerosis.
